# Exploring the Role of Large Language Models in Melanoma: A Systematic Review

**DOI:** 10.3390/jcm13237480

**Published:** 2024-12-09

**Authors:** Mor Zarfati, Girish N. Nadkarni, Benjamin S. Glicksberg, Moti Harats, Shoshana Greenberger, Eyal Klang, Shelly Soffer

**Affiliations:** 1 Department of Internal Medicine, Soroka University Medical Center, Beer-Sheva 84101, Israel; zarfatimor@gmail.com; 2 Division of Data-Driven and Digital Medicine, Department of Medicine, Icahn School of Medicine at Mount Sinai, New York, NY 10029, USA; girish.nadkarni@mountsinai.org (G.N.N.); ben.glicksberg@gmail.com (B.S.G.); 3 Sheba Medical Center, Department of Plastic and Reconstructive Surgery, Ramat-Gan 52621, Israel; harats@gmail.com; 4 Faculty of Medicine, Tel Aviv University, Tel Aviv 52621, Israel; shoshana.greenberger@sheba.health.gov.il (S.G.); soffer.shelly@gmail.com (S.S.); 5 Pediatric Dermatology Unit, Sheba Medical Center, Department of Dermatology, Ramat Gan 52621, Israel; 6 Institute of Hematology, Davidoff Cancer Center, Rabin Medical Center, Petah-Tikva 49100, Israel

**Keywords:** large language models, melanoma, artificial intelligence

## Abstract

**Objective**: This systematic review evaluates the current applications, advantages, and challenges of large language models (LLMs) in melanoma care. **Methods:** A systematic search was conducted in PubMed and Scopus databases for studies published up to 23 July 2024, focusing on the application of LLMs in melanoma. The review adhered to PRISMA guidelines, and the risk of bias was assessed using the modified QUADAS-2 tool. **Results:** Nine studies were included, categorized into subgroups: patient education, diagnosis, and clinical management. In patient education, LLMs demonstrated high accuracy, though readability often exceeded recommended levels. For diagnosis, multimodal LLMs like GPT-4V showed capabilities in distinguishing melanoma from benign lesions, but accuracy varied, influenced by factors such as image quality and integration of clinical context. Regarding management advice, ChatGPT provided more reliable recommendations compared to other LLMs, but all models lacked depth for individualized decision-making. **Conclusions:** LLMs, particularly multimodal models, show potential in improving melanoma care. However, current applications require further refinement and validation. Future studies should explore fine-tuning these models on large, diverse dermatological databases and incorporate expert knowledge to address limitations such as generalizability across different populations and skin types.

## 1. Introduction

Large language models (LLMs), including ChatGPT, Gemini, and Llama, are artificial intelligence (AI) models designed to understand and generate human-like text [1]. These models are gaining recognition across various medical specialties for their potential to assist with clinical tasks [2,3,4,5,6,7]. However, their specific role in dermatology, particularly in melanoma care, remains under investigation [8]. Multimodal LLMs, such as GPT-4 Vision (GPT-4V), further expand this potential by combining visual and textual data. This capability could improve applications in medical imaging and diagnosis [9].

Melanoma, the most aggressive form of skin cancer, is responsible for more than 80% of skin cancer mortality [10]. Early melanoma detection enables successful surgical treatment and significantly better survival rates. Once metastasis occurs, prognosis worsens considerably. Therefore, timely and accurate diagnosis is critical for patient outcomes [11]. Clinically, melanoma is often identified by the ABCDE rule, which evaluates asymmetry, border irregularity, color variation, diameter, and evolving characteristics of skin lesions [10]. Current treatment approaches include surgical excision, targeted therapy, immunotherapy, and radiation therapy, with treatment selection based on stage and molecular profile [11].

Previous studies have shown mixed results regarding the use of LLMs in dermatology, leading to caution among dermatologists [12]. However, these tools, when properly optimized, may enhance melanoma diagnosis, patient communication, and treatment outcomes.

This systematic review aims to evaluate the current applications, advantages, and challenges associated with the use of LLMs in melanoma care.

## 2. Foundational Concepts

**Figure 1:**
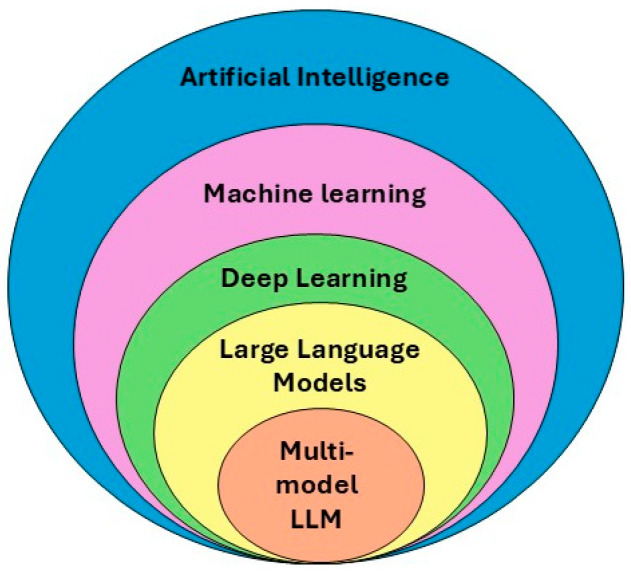
Hierarchy diagram of artificial intelligence (AI) terms.

Below are the key concepts related to LLMs and their applications in healthcare. In Figure 1, we present a hierarchy diagram of AI terms.

### 2.1. Artificial Intelligence and Deep Learning

AI refers to the development of algorithms capable of performing tasks that typically require human intelligence. Machine learning (ML), a key driver of recent AI advances, enables systems to learn from data rather than following fixed rules. Deep learning is a subset of ML that employs artificial neural networks to analyze different types of data and learn from them. Examples include language comprehension and image pattern recognition [13,14].

### 2.2. Artificial Neural Networks

Artificial neural networks form the foundation of deep learning. Inspired by biological neural networks, they consist of interconnected nodes, or “neurons”, organized in layers. Each neuron receives inputs, processes them, and passes an output to the subsequent layer. Each neuron is a simple computational unit, similar to a single logistic regression function. By adjusting the connections between neurons based on the input data, neural networks can learn to recognize patterns and generate predictions [13].

### 2.3. Large Language Models

LLMs are large deep learning models that process and generate human-like text. Composed of multiple transformer layers, these models employ an attention mechanism to selectively focus on different parts of the input data. This structure allows them to excel in tasks such as text recognition, language translation, and content generation [15]. Notable examples of LLMs include ChatGPT by OpenAI and LLaMA by Meta [16,17,18].

### 2.4. Convolutional Neural Networks

CNNs represent a specialized deep learning architecture optimized for visual analysis. These self-learning algorithms process images through multiple layers, each extracting features like edges, textures, and patterns. Each layer applies mathematical filters (convolutions) to detect specific visual elements, enabling image recognition capabilities [19].

### 2.5. Multimodal Large Language Models

Multimodal LLMs (MLLMs) extend the capabilities of traditional LLMs by incorporating multiple other data modalities into text, such as images, sound, and videos. MLLMs are typically built as foundation models—large-scale, general-purpose models pre-trained on massive multimodal datasets. These models provide a base that can be fine-tuned for specific tasks.

Vision Language Models (VLMs) are a subset of multimodal models specifically focusing on image–text interactions. Unlike VLMs, MLLMs are more versatile and can handle broader multimodal reasoning tasks that involve multiple data types such as audio, video, and structured data.

The architecture of MLLMs typically consists of three key components: modality-specific encoders (processing individual data types), a central LLM backbone (processes text and handles core reasoning), and modality interfaces (unify these inputs into a shared representation) [20].

The field of MLLMs has evolved rapidly, from early models like Flamingo [21] (2022) [21] to advanced systems like GPT-4V (2023) [22] and the latest generation including Claude 3 and Gemini 1.5 and GPT-4o (2024) [10].

## 3. Materials and Methods

### 3.1. Search Strategy

A systematic review was conducted according to the Preferred Reporting Items for Systematic Reviews and Meta-Analyses statement (PRISMA) guidelines and the recommendations for systematic reviews of prediction models (CHARMS checklist) [23,24]. The study is registered with PROSPERO (CRD42024575859, link: https://www.crd.york.ac.uk/prospero/display_record.php?RecordID=575859, accessed on 3 October 2024) [25].

We searched the literature for applications of LLMs in melanoma using PubMed and Scopus. A systematic search of the published literature was conducted on 23 July 2024. Our search query was “((“Melanoma”) AND ((“ChatGPT”) OR (“large language models”) OR (“OpenAI”) OR (“Microsoft Bing”) OR (“google bard”) OR (“google gemini”)))”. We included Microsoft Bing in our search strategy, although it is not an LLM itself, as it incorporates LLM technology. The term “MLLM” (multimodal large language model) was not included in our initial search strategy. References to multimodal language models emerged from papers identified through our original search terms related to LLMs in melanoma care. To ensure thoroughness, we also reviewed the reference lists of relevant articles, but this did not yield any additional studies that met the inclusion criteria.

We excluded articles that did not specifically evaluate the application of LLMs in melanoma, non-original articles, and conference abstracts.

### 3.2. Study Selection

The titles and abstracts of the identified studies were screened to determine their eligibility based on the inclusion and exclusion criteria. Any uncertainty was resolved through discussion between two reviewers, with a third reviewer consulted when necessary. The full texts of the selected articles were then independently assessed by two reviewers (MZ, SS). Discrepancies were resolved through consensus or consultation with a third reviewer (EK).

### 3.3. Data Extraction

Data extraction was conducted using a standardized form to ensure consistency. Key information extracted included the first author’s name, year of publication, sample size, LLM model types, objectives, and main findings.

To investigate the specific applications and effectiveness of LLMs in different aspects of melanoma care, we divided the articles into three subgroups: patient education, clinical management, and diagnosis.

### 3.4. Quality Assessment and Risk of Bias

To evaluate the risk of bias, we used the adapted version of the Quality Assessment of Diagnostic Accuracy Studies criteria (QUADAS-2) [26].

## 4. Results

**Figure 2:**
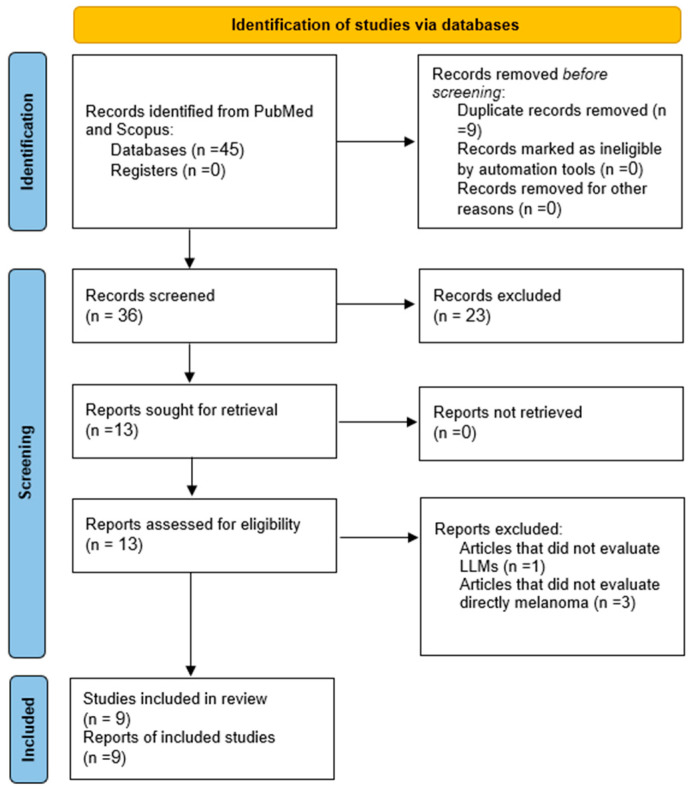
Flow diagram of the search and inclusion process based on the Preferred Reporting Items for Systematic Reviews and Meta-Analyses (PRISMA) guidelines.

Our literature search yielded a total of 45 articles from PubMed and Scopus. After the removal of nine duplicates, the screening process found nine studies that met our inclusion criteria. We did not identify additional studies via reference screening [27,28,29,30,31,32,33,34,35]. The process of study selection and the screening methodology are detailed in the PRISMA flow chart (Figure 2).

According to the QUADAS-2 tool, most papers scored as having a low to moderate risk of bias for the interpretation of the index test. A detailed assessment of the risk of bias is provided in Table 1.

The characteristics of the studies are presented in Table 2. A summary of the objective, sample size, reference standard, main findings, and conclusions are presented in Table 3. The main advantages and challenges in the included studies are presented in Table 4.

Of the nine studies, five were comparative, evaluating and comparing various LLM models, such as ChatGPT, BARD, and BingAI [27,29,30,33,34]. The remaining four studies focused on a single LLM, specifically different versions of ChatGPT [28,31,32,35]. Three studies specifically examined multimodal LLMs, such as GPT-4V and LLaVA, highlighting their unique capabilities and associated challenges [29,31,33].

The included studies were diverse in their objectives, methodologies, and evaluation metrics. The studies focused on the application of LLMs in melanoma diagnosis, patient education, and clinical decision-making.

### 4.1. Patient Education

Four studies evaluated the use of LLMs in patient education, focusing on the accuracy of responses to common patient questions [28,30,34,35]. ChatGPT 4.0 and ChatGPT 3.5 were noted for their relatively high accuracy.

Deliyannis et al. found that while both ChatGPT and BARD can generate accurate educational responses, both ChatGPT 4.0 and 3.5 outperformed BARD [30]. Anguita et al. focused on choroidal melanoma and found no significant accuracy differences between ChatGPT 3.5, Bing AI, and DocsGPT beta [34].

Young et al. reported that ChatGPT 4.0 generates mostly accurate responses, scoring 4.9/5. However, only 64% of these responses were considered suitable for patient use, indicating that ChatGPT may be more effective as a supplemental tool in clinical practice. The study also found that the average readability score corresponded to a college-level comprehension, suggesting that the content might be too advanced for public use [35].

Roster et al. addressed this readability issue by evaluating ChatGPT’s responses to questions about sunscreen and melanoma from the American Academy of Dermatology’s (AAD) website. They investigated whether prompt engineering techniques (strategic prompting) could improve readability. The study compared ChatGPT’s responses after two rounds of strategic prompting with the original answers from the AAD website. The findings showed that the initial prompt did not lower the reading level compared to the AAD content. However, with additional prompting, the reading level was reduced to 7th grade, compared to the AAD’s 9th-grade level. This suggests that with proper prompt engineering, LLMs could improve the readability of medical information for melanoma patients [28].

### 4.2. Melanoma Diagnosis

Four studies examined the use of LLMs in melanoma diagnosis, focusing on their ability to identify and classify melanoma using clinical and dermoscopic data [29,31,32,33]. Multimodal LLMs, such as GPT-4V and LLaVA, played a key role in the majority of these evaluations.

Cirone et al. assessed GPT-4V and LLaVA, emphasizing their ability to integrate visual and textual data. The study used macroscopic images of melanoma and melanocytic nevi obtained from the MClass-D dataset. The prompts varied in their specificity, with some being general and asking for descriptions of the images, while others addressed the ABCDE features of melanoma. Some prompts also assessed the effects of background skin color on predictions. GPT-4V demonstrated superior performance, with an overall accuracy of 85%, compared to 45% for LLaVA. Notably, GPT-4V consistently provided descriptions of relevant ABCDE features and accurately identified melanoma. Also, LLaVA had difficulty recognizing melanoma in skin of darker color, unlike GPT-4V [33]. This finding is consistent with those of Akrout et al., who also showed that GPT-4V outperformed LLaVA across all assessed features, though both models require further refinement to enhance diagnostic accuracy [29].

Shifai et al. evaluated ChatGPT Vision’s diagnostic accuracy in identifying melanoma using dermoscopic images from the ISIC archives. The model provided three ranked differential diagnoses for 100 melanocytic lesions. The sensitivity, specificity, and diagnostic accuracy varied depending on whether the top diagnosis or the top three diagnoses were considered [31].

These findings suggest that ChatGPT Vision may not yet be suitable for independent clinical use without additional refinement.

### 4.3. Management Advice

Only one study specifically evaluated the use of LLMs in providing melanoma management advice. Mu et al. conducted a comparative analysis of several LLMs (ChatGPT 4.0, BARD, and BingAI) to assess their performance in this context. The study used five prompts related to melanoma management. ChatGPT 4.0 consistently provided more reliable, evidence-based clinical advice, outperforming the other models, with significant differences noted compared to BARD and marginal differences compared to BingAI. However, none of the models evaluated the risks and benefits associated with their recommendations. The limited number of questions restricts the generalizability of the findings [27].

## 5. Discussion

This review’s findings underscore the potential of LLMs across various domains in melanoma care, including patient education, disease diagnosis, and management advice. Of particular interest is the emergence of multimodal LLMs, which integrate visual and textual data to address the complexities of medical imaging and clinical decision-making.

In patient education, LLMs demonstrated an ability to generate accurate and readable responses to common melanoma-related queries. For example, Roster et al. showed that strategic prompting can enhance the readability of ChatGPT’s outputs [28]. This finding suggests that with appropriate fine-tuning, LLMs could become valuable tools for creating accessible patient education materials, enabling individuals to make informed decisions.

In melanoma diagnosis, multimodal LLMs such as GPT-4V and LLaVA exhibited capabilities in distinguishing melanoma from benign lesions. Cirone et al. and Akrout et al. demonstrated GPT-4V’s superior performance [29,33], particularly in handling variations in skin tone and image manipulations [33]. Zhou et al. presented SkinGPT-4, a multimodal LLM trained on a large collection of skin disease images and clinical notes. SkinGPT-4 demonstrated the ability to accurately diagnose various skin conditions and provide interactive treatment recommendations [36]. In addition to LLMs, AI-based methods, particularly those utilizing dermoscopic images, have shown promising results in assisting with melanoma detection. A systematic review by Patel et al. found that AI-based algorithms achieved a higher ROC (>80%) compared to dermatologists in the detection of melanoma using dermoscopic images [37]. However, it is important to recognize that multimodal LLMs are not yet reliable for independent clinical use. Their performance may be influenced by factors such as dataset limitations, image quality, and the lack of clinical context.

Despite these limitations, multimodal LLMs may hold promise for applications in medical education. Sorin et al. explored the potential of multimodal LLMs in ophthalmology education, suggesting that they could significantly impact this field by providing detailed explanations of ocular examination and imaging findings [38]. Similarly, in the context of melanoma and dermatology, multimodal LLMs could assist students in identifying and describing lesion characteristics, considering differential diagnoses, and developing their clinical reasoning skills.

Mu et al. investigated the use of LLMs for management advice and found that ChatGPT provided more reliable and evidence-based recommendations compared to BARD and BingAI. However, all models were limited by a lack of depth and specificity, reducing their utility in individualized clinical decision-making [27]. This finding emphasizes the need for further refinement and validation of LLMs to ensure that their recommendations align with clinical guidelines.

The limitations of this review include the small number of studies, heterogeneity in methodologies, and variations in evaluation metrics. Additionally, most studies had small sample sizes and did not involve patients in the question selection process. Furthermore, most studies focused on general melanoma questions rather than specific clinical scenarios.

## 6. Conclusions

This review highlights the potential of LLMs, particularly multimodal models, in improving melanoma care through patient education, diagnosis, and management advice. While these technologies show promise, they remain assistive tools that complement, rather than substitute, medical expertise. Despite promising results, current LLM applications require further refinement to ensure clinical utility, and their use should always be under physician supervision. Future studies should explore fine-tuning these models on large dermatological databases and incorporate expert knowledge.

## Figures and Tables

**Table 1:** Risk of bias according to the QUADAS-2.

	Patient Selection	Index Test	Reference Standard	Flow and Timing	Overall Bias
Young J. N. [35]	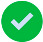	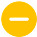	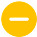	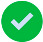	Low to moderate
Anguita R. [34]	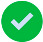	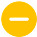	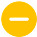	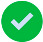	Low to moderate
Deliyannis E. P. [30]	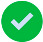	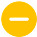	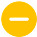	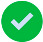	Low to moderate
Roster K. [28]	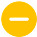	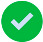	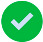	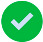	Low to moderate
Cirone K. [33]	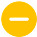	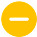	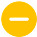	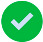	Moderate
Shifai N. [31]	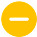	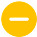	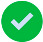	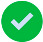	Low to moderate
Akrout M. [29]	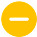	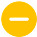	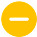	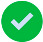	Moderate
Karampinis E. [32]	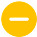	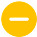	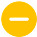	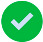	Moderate
Mu X. [27]	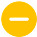	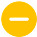	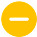	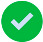	Moderate

Judgement: 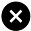 high risk, 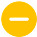 moderate risk, 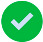 low risk.

**Table 2:** Details about reviewed articles.

Group	Title	First Author	Journal	Year
Patient education	The utility of ChatGPT in generating patient-facing and clinical responses for melanoma	Young J. N. [35]	*Journal of the American Academy of Dermatology*	2023
	Assessing large language models’ accuracy in providing patient support for choroidal melanoma	Anguita R. [34]	*Eye (Lond)*	2024
	Comparative performance analysis of ChatGPT 3.5, ChatGPT 4.0 and Bard in answering common patient questions on melanoma	Deliyannis E. P. [30]	*Clinical and Experimental Dermatology*	2024
	Readability and Health Literacy Scores for ChatGPT-Generated Dermatology Public Education Materials: Cross-Sectional Analysis of Sunscreen and Melanoma Questions	Roster K. [28]	*JMIR Dermatology*	2024
Melanoma Diagnosis	Assessing the Utility of Multimodal Large Language Models (GPT-4 Vision and Large Language and Vision Assistant) in Identifying Melanoma Across Different Skin Tones	Cirone K. [33]	*JMIR Dermatology*	2024
	Can ChatGPT Vision Diagnose Melanoma? An Exploratory Diagnostic Accuracy Study	Shifai N. [31]	*Journal of the American Academy of Dermatology*	2024
	Evaluation of Vision LLMs GTP-4V and LLaVA for the Recognition of Features Characteristic of Melanoma	Akrout M. [29]	*Journal of Cutaneous Medicine and Surgery*	2024
Diagnosis of melanoma and medical education	Can Artificial Intelligence “Hold” a Dermoscope? The Evaluation of an Artificial Intelligence Chatbot to Translate the Dermoscopic Language	Karampinis E. [32]	*Diagnostics (Basel)*	2024
Management advice	Comparison of large language models in management advice for melanoma: Google’s AI BARD, BingAI and ChatGPT	Mu X. [27]	*Skin Health*	2023

**Table 3:** A summary of the reviewed articles.

First Author	Model Used	Objective	Reference Standard	Sample Size	Main Findings	Conclusion
Young J. N. [35]	ChatGPT 4.0	Assess the appropriateness, clinical applicability, accuracy, and readability of ChatGPT 4.0 responses to melanoma-related questions.	Three board-certified dermatologists	25 melanoma- related patient questions	Accuracy: 4.88/5 with agreement (80%, Fleiss K coefficient 0.808, *p* < 0.001). Appropriateness: 92% Sufficiency: 64% Readability: Average FRES 42.67 (college-level readability)	ChatGPT 4.0 generates mostly accurate, but not sufficient, responses to melanoma patient questions, but it presents it at a level to advanced for public use.
Anguita R. [34]	ChatGPT 3.5, Bing AI, DocsGPT beta	Evaluate the accuracy of information provided by LLMs in response to common questions about choroidal melanoma.	Three ocular oncology experts	27 questions: 12 medical advice and 15 pre- and post-operative advice	Medical advice questions: Accuracy: GPT 3.5 92%, Bing AI 58%, DocsGPT 58% Pre and post-operative advice: Accuracy: GPT 3.5 86%, Bing AI 86%, DocsGPT 73%. 57% of responses varied across triplicated queries (Cohen’s kappa = 0.43, *p* < 0.05)	The three models demonstrate accuracy in response to most patient questions. There are no significant differences between the models.
Deliyannis E. P. [30]	ChatGPT 3.5, ChatGPT 4.0, Google Bard	Evaluate and compare the accuracy, readability, comprehensiveness and reproducibility of responses provided by ChatGPT 3.5, ChatGPT 4.0, and Google Bard to common melanoma patient questions.	A consultant dermatologist and a senior dermatology trainee	205 questions identified; 22 questions selected	Total score for all 4 parameters, readability, comprehensiveness, reproducibility (out of 5): ChatGPT 3.5: 4.51, 4.68, 4.38, 4.41 ChatGPT 4.0: 4.43, 4.65, 4.4, 4.2 Bard: 4.14, 4.35, 4.09, 3.89 ChatGPT 3.5 and 4.0 consistently scored higher than Bard for all parameters.	ChatGPT and BARD may generate educational responses to common patient queries. Both versions of ChatGPT outperform BARD.
Roster K. [28]	ChatGPT	Evaluate the readability of ChatGPT-generated public education dermatology materials on sunscreen and melanoma, and to determine if strategic prompting can improve readability to meet the American Medical Association (AMA) guidelines (6th-grade reading level or less).	Readability was compared to AAD. Accuracy was evaluated by three dermatology residents. The study evaluated initial ChatGPT responses and responses after two rounds of strategic prompting.	42 prompts, sourced from the American Academy of Dermatology (AAD) website’s frequently asked questions (FAQs).	Melanoma FAQ Readability: (FRES score, average grade) AAD: 56.2, 9th grade ChatGPT initial: 46.5,10th grade ChatGPT with 2 prompt: 58.9, 8th grade ChatGPT with 3 prompts: 59.3, 7th grade Prompting lowered the reading level vs. AAD (for 3 prompts *p* = 0.007) Melanoma FAQs accuracy (scale from 1 to 3): AAD: 2.82 ChatGPT initial: 2.89 ChatGPT with 2 prompt: 2.63 ChatGPT with 3 prompts: 2.62	Using strategic prompting, ChatGPT could be used to enhance readability of medical data for melanoma patients. This prompting may result in less accuracy.
Cirone K. [33]	GPT-4V, LLaVA	Assess the ability of LLMs, specifically GPT-4 Vision and LLaVA, to accurately recognize and differentiate between melanoma and benign melanocytic nevi across different skin tones.	Macroscopic images of melanoma and melanocytic nevi obtained from the MClass-D dataset.	20 text-based prompts, each tested on 3 images, resulting in 60 unique image–prompt combinations.	GPT-4V Performance: Overall accuracy: 85% Consistently provided descriptions of relevant ABCDE features. Accurately identified melanoma across different skin tones and -recognized alterations in images. LLaVA Performance: Overall accuracy: 45% Unable to confidently identify melanoma in individuals with darker skin tones. Vulnerable to visual prompt injection and manipulation, leading to diagnostic errors.	GPT-4V and LLaVA show potential in identifying melanoma across different skin tones, but further refinement is needed. GPT-4V outperforms LLaVA in overall accuracy.
Shifai N. [31]	ChatGPT Vision	Assess the diagnostic accuracy of ChatGPT Vision in identifying melanoma using dermoscopic images.	Dermoscopy images from ISIC archives.	100 melanocytic lesions (50 melanomas and 50 benign nevi)	The model provided 3 ranked differential diagnoses. Top Diagnosis: Sensitivity: 32% Specificity: 40% Diagnostic accuracy: 36% Top-3 Differential Diagnoses: Sensitivity: 56% Specificity: 53.3% Diagnostic accuracy: 54.7% Malignant vs. Benign (Top Diagnosis): Sensitivity: 46% Specificity: 78% Diagnostic accuracy: 62% Malignant vs. Benign (Top-3 Diagnoses): Sensitivity: 78% Specificity: 46.7% Diagnostic accuracy: 62.3%	ChatGPT Vision’s current capabilities are inadequate for reliable melanoma diagnosis.
Akrout M. [29]	GTP-4V, LLaVA	Assess the ability of vision LLMs to recognize, classify, and appropriately comment on the ABCDE features of melanoma lesions.	Macroscopic images obtained from the publicly available MD-class dataset and Dermnet NZ.	55 unique text-based prompts consisting of questions and instructions, and image-based prompts highlighting areas of focus	GTP-4V Performance: Accurately described asymmetry, border, color, diameter, and evolution. Inconsistently identified melanoma subtypes Vulnerable to visual prompt injections. LLaVA Performance: Accurately described asymmetry, border, and color. Inaccurately assessed diameter and evolution. Inconsistently identified melanoma subtypes. Less vulnerable to visual prompt injections.	GTP-4V outperformed LLaVA. While GTP-4V and LLaVA show promise in recognizing features characteristic of melanoma, both models require further refinement to improve diagnostic accuracy and consistency.
Karampinis E. [32]	ChatGPT 3.5	Assess the clarity of dermoscopic language translated by an AI chatbot and its role in facilitating accurate diagnoses and educational opportunities for novice dermatologists.	30 participants with a certification in dernoscopy	The survey comprised instances of dermoscopic descriptions, including 3 pigmented lesions (1 melanoma and 2 nevi)	Pigmented lesion scores (scale of 1 to 3): Completeness: 2.4 ± 0.88 Helpful in diagnosis: 2.8 ± 0.48 Teaching tool: 2.7 ± 0.59 For pigmented lesions, incorporating clinical patient data did not significantly change the results.	AI chatbot demonstrates potential in translating dermoscopic language but requires further development to improve its accuracy and reliability for clinical use.
Mu X. [28]	ChatGPT-4, BingAI, Google’s AI BARD	Compare the performance of Google’s AI BARD, BingAI, and ChatGPT-4 in providing melanoma management advice based on current clinical guidelines and the literature.	2 plastic surgent residents, 1 registrar and 3 specialist plastic surgeons	5 questions on melanoma management	Readability (Flesch Reading Ease Score, Flesch–Kincaid Grade Level): ChatGPT: 35.42, 11.98 BARD: 32.1, 15.03 BingAI: 29.88,13.58 The mean readability exhibited considerable similarity. Reliability: DISCERN score: ChatGPT 58 (+-6.44) BARD 36.2 (+-34.06) BingAI’s 49.8 (+-22.28). The only statistically significant test was comparing ChatGPT to BARD for the DISCERN score (*p*-value 0.04).	ChatGPT provides more reliable, evidence-based clinical advice than BARD and BingAI. However, all models lack depth and specificity, limiting their use in individualized clinical decision-making.

**Table 4:** Advantages and challenges of the reviewed articles.

First Author	Model Used	Challenges
Young J. N. [35]	The responses were evaluated by three board-certified dermatologists, ensuring that the assessment of the AI’s performance was thorough and conducted by knowledgeable professionals. The agreement between the evaluators was statistically significant.	Patients were not involved in the question selection process, potentially missing out on patient perspectives.
Anguita R. [34]	The study compares 3 different LLMs, offering a broad perspective on their performance. The study relies on the assessment of three experts who were blinded to the LLM they were using.	The study is limited to a subtype of melanoma. The study focussed only on accuracy and did not evaluate other aspects.
Deliyannis E. P. [30]	Questions were identified from online sources such as Facebook groups, national foundations, and charity websites, increasing the relevance and practical importance of the questions evaluated. The study compares three different LLMs, offering a broad perspective on their performance. The responses were assessed for accuracy, readability, comprehensiveness, and reproducibility, providing a thorough evaluation.	Only 2 assessors were involved in scoring the responses, which might limit the robustness of the evaluation. Readability was not assessed using FRES score.
Roster K. [28]	The use of multiple readability and health literacy tools provides a thorough evaluation of the text readability. Accuracy was assessed by 3 dermatology residents, ensuring the reliability of the content evaluation. The use of multiple prompts on the same FAQ demonstrates the model’s strength in improving readability.	The study only evaluates ChatGPT, limiting the comparison with other LLMs. It is unclear how many prompts specifically addressed melanoma.
Cirone K. [32]	The use of Multiple LLMs offers a broad perspective on their performance. Evaluation of the models’ ability to handle image manipulations and consider skin tone variations demonstrates the models’ effectiveness across different diagnostic factors.	Absence of statistical significance tests. The number of benign nevi vs. melanomas that were recognized or un-recognized is not specified. Thus, the reader cannot interpret the sensitivity and specificity of the diagnosis. The study does not specify the number of evaluators who assessed the accuracy of the results, as well as the unknown proficiency of these evaluators.
Shifai N. [31]	The study uses a balanced dataset with an equal number of melanomas and benign nevi, thus improving the credibility of the study. The evaluation uses sensitivity and specificity metrics to assess the model’s diagnostic performance for both positive and negative cases.	The absence of intermediate melanocytic lesions, such as dysplastic nevi, oversimplifies the evaluation compared to routine clinical settings. Factors such as anatomic site, skin type, nevi subtype, melanoma subtype, and tumor thickness were not considered in the analyses.
Akrout M. [29]	The study utilized a balanced dataset covering various melanoma stages which enhances the robustness of the evaluation. The evaluation included metrics for describing ABCDE features, identifying melanoma subtypes, and handling visual prompt injections, offering a detailed assessment of model performance. The use of Multiple LLMs offers a broad perspective on their performance.	No statistical tools were used. The study utilized “textbook” or idealized images of melanoma, which may not accurately represent the diverse range of lesions encountered in real-world clinical settings. The evaluators’ identities and their proficiency in interpreting the model outcomes are unknown.
Karampinis E. [32]	The results are based on feedback from 30 participants, providing diverse insights into the chatbot’s performance. The prompts were evaluated both with and without incorporating additional clinical patient data.	Only three descriptions of pigmented lesions were used. The study did not focus specifically on melanotic lesions.
Mu X. [21]	The study involves a panel of experienced board-certified plastic surgeons to assess the responses. The use of multiple readability matrixes provides a thorough evaluation of the text readability. The comparison of multiple LLMs offers a broad perspective on their performance.	The small number of questions limits the generalizability of the results. The questions examined were mostly general and did not address a patient’s clinical background. The study evaluates LLMs’ responses based solely on existing guidelines, without considering newer research that may provide more up-to-date information.

## Data Availability

No new data were created or analyzed in this study. Data sharing is not applicable to this article.
